# Localization and characterization of cutaneous neurogenic inflammation in acute gastric mucosal injury in rats: A possible morphological explanation for visceral sensitization?

**DOI:** 10.1371/journal.pone.0324136

**Published:** 2025-06-04

**Authors:** Shi-yi Qi, Jin-wen Lin, Shi-hao Wang, Li-li Lin, Shen Lin, Jian-guo Chen, You-cong Ni, Xin Du, Ling-Ling He, Xin Wu, Dong Lin

**Affiliations:** 1 College of Acupuncture, Fujian University of Traditional Chinese Medicine, Fuzhou, Fujian, China; 2 The Second People’s Hospital Affiliated to Fujian University of Traditional Chinese Medicine, Fuzhou, Fujian, China; 3 Fujian Maternity and Child Health Hospital, Fuzhou, Fujian, China; 4 Department of Electrical Engineering and Automation, Fuzhou University, Fuzhou, Fujian, China; 5 School of Computer and Cyberspace Security, Fujian Normal University, Fuzhou, Fujian, China; 6 Department of Rehabilitation, The Third People’s Hospital Affiliated to Fujian University of Traditional Chinese Medicine, Fuzhou, Fujian, China; Helwan University, EGYPT

## Abstract

This investigation transcends traditional methodologies by providing a quantitative analysis of the dynamic relationship between visceral pathologies and neurogenic spots, employing an acute gastric mucosal injury (AGMI) rat model to map the somatotopic distribution of visceral sensitization. Through hydrochloric acid-induced plasma extravasation and Evans Blue dye (EB) marking, coupled with a geospatial grid system and multivariate statistical analysis, we identified Feature Regions (FRs) with distinct neurogenic responses. Notably, the right T10-13 dermatomere, or FR-11’, exhibited elevated levels of nociceptive neuropeptides and serotonin, indicative of its significant role in pain perception. The application of electroacupuncture at FR-11’ revealed enhanced therapeutic outcomes compared to the conventional acupoint BL-21, positioning it as a promising modality for the management of visceral pain. These findings contribute substantially to our understanding of the mechanisms underlying visceral-somatic pain and pave the way for innovative pain management interventions in clinical settings.

## 1 Introduction

Head, in 1893, delineated distinct somatic referred areas associated with various visceral diseases, a phenomenon now known as Head’s zones [1], which are characterized by the manifestation of referred pain on the external body surface in response to specific visceral disorders. This intricate process is fundamentally linked to the central sensitization of the spinal cord and the initiation of neurogenic inflammation [2–4]. The transmission of nociceptive sensory signals from visceral organs culminates in the formation of hyperresponsive areas on the skin, termed neurogenic spots. These are resultant of cutaneous neurogenic inflammation within the dermatome that overlaps with visceral afferent innervations [2,5]. Visualization of these phenomena can be facilitated experimentally through the injection of Evans blue dye (EB) [2,6–8].

Additionally, acupuncture, a medical practice with its origins in China over 2500 years ago [9], has garnered substantial recognition for treating various ailments, particularly visceral and pain-related conditions [10–12]. Central to this practice is the strategic insertion of fine needles into acupoints—specific anatomical sites on the body surface integral to acupuncture’s therapeutic effects. Despite extensive research, the underlying structures of acupoints remain elusive [13]. Notably, it is postulated that cutaneous neurogenic inflammatory spots have a connection with acupoints [14–17], which show significant convergence with pain-sensitive regions during the manifestation of visceral diseases [18,19]. These sensitized acupoints are predominantly characterized by enhanced sensory responsiveness [20], expanded range [21], and increased functionality [22].

However, the investigation of cutaneous neurogenic inflammation presents a primary challenge, which involves the elucidation of the governing law of these discrete phenomena, the identification of disease-related points, and the unravelling of their inherent attributes. This challenge is especially significant considering that these points, particularly those denoted by EB dye extravasation, are isolated incidents, infrequently shared between two rats in terms of both location and number. To date, these spots have been primarily identified via direct observation or basic frequency analysis [23,24], yet the observational results often lack quantification. This lack of precision introduces an inherent degree of randomness and uncertainty, potentially leading to confusion.

**Fig 1:**
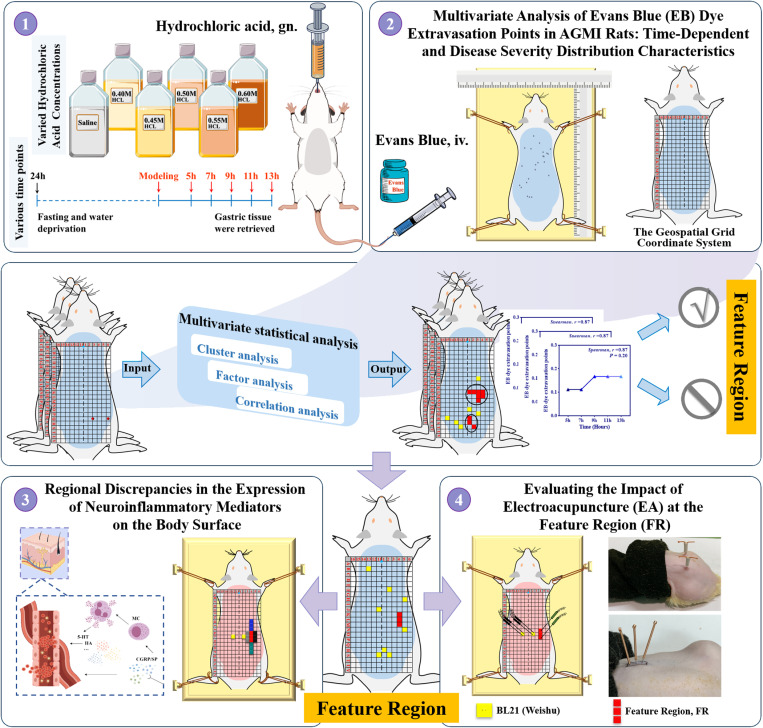
Study Overview Schematic. Schematic representation delineating the experimental design, key interventions, and analytical approaches employed in the investigation of acute gastric mucosal injury in rats.

In this investigation, we extend our prior work that demonstrated mustard oil-induced visceral sensitization after colitis [25] and the formation of cutaneous neurogenic inflammatory spots, identifiable via established coordinate systems. Our current study aims to elucidate the distribution of visceral sensitization on the body surface and to further comprehend the biological foundations of these patterns, assessing their potential for intervention. We hypothesize a direct correlation between visceral disease states and body surface alterations, with a lack of such a relationship suggesting independence of these phenomena. Employing a suite of experimental approaches, we initially conducted a multivariate analysis of Evans Blue dye extravasation in acute gastric mucosal injury (AGMI) rats to define a Feature Region (FR) reflective of temporal dynamics and disease severity. We then probed for regional disparities in neuroinflammatory mediator expression within the FR, contrasting these findings against adjacent areas and established acupoints. The concluding phase evaluated electroacupuncture’s efficacy at the FR in modulating AGMI-induced pathologies (**Fig 1**).

## 2 Materials and methods

### 2.1 Animals

In our study, female Wistar rats, aged 6 weeks and weighing 185-195g, were sourced from the Laboratory Animal Center at Fujian University of Traditional Chinese Medicine. They were housed under controlled conditions with a stable temperature of 22 ± 1 °C and a 12-hour light-dark cycle, with free access to food and water. The facility’s operation was licensed under SYXK (Min) 2019–0007. Our experimental procedures, sanctioned by the Ethics Committee of Fujian University of Traditional Chinese Medicine (Permit Number: FJTCM IACUC2022127), complied with the Ministry of Science and Technology of the People’s Republic of China’s 2006 Guidelines for the Proper Care of Experimental Animals. About the effect of sex selection on the distribution, The selection of a single gender aimed to minimize variability and enhance experimental consistency.This is based on the our previous research basis [26].

### 2.2 AGMI rats model establishment

Prior to the initiation of the experiments, the rats underwent a 24-hour fasting period, while water was available ad libitum. Hydrochloric acid (HCl) solutions of varying concentrations (0.40 M, 0.45 M, 0.50 M, 0.55 M, 0.60 M), or saline as a control, were freshly prepared and administered at a dosage of 1 mL/100 g of body weight [23] using a 16-gauge gavage needle (Kent Scientific Corporation, Torrington, CT, USA). The replication of grabbing actions in rats from both the blank and control groups was considered crucial.

### 2.3 Neurogenic inflammation detection in skin via Evans blue dye injection

Four hours post-HCl administration, rats were anesthetized using inhalation anesthetics (isoflurane). Subsequent to this, an EB dye solution (Sigma Chemical Co, St. Louis, MO), prepared in sterile water (20 mg/kg), was intravenously introduced via the tail vein, with successful injection indicated by the immediate bluing of the rat’s eyes [2,7,8]. Prior to and during the hair removal process, precautions were taken to prevent skin injuries. The dorsal hair was thoroughly removed using a commercially available hair removal cream. For precise positioning, the rats were immobilized in a standardized prone posture on the rat plate, with the tripod adjusted to sustain a constant 40 cm elevation from the rat plate. Following this, images of the rats’ dorsal region were subsequently captured at 5, 7, 9, 11, and 13 hours post-model establishment using a camera. During these observation phases, the rats were maintained under inhalation anesthesia with isoflurane.

### 2.4 Positioning of dorsal region in rats

**Fig 2:**
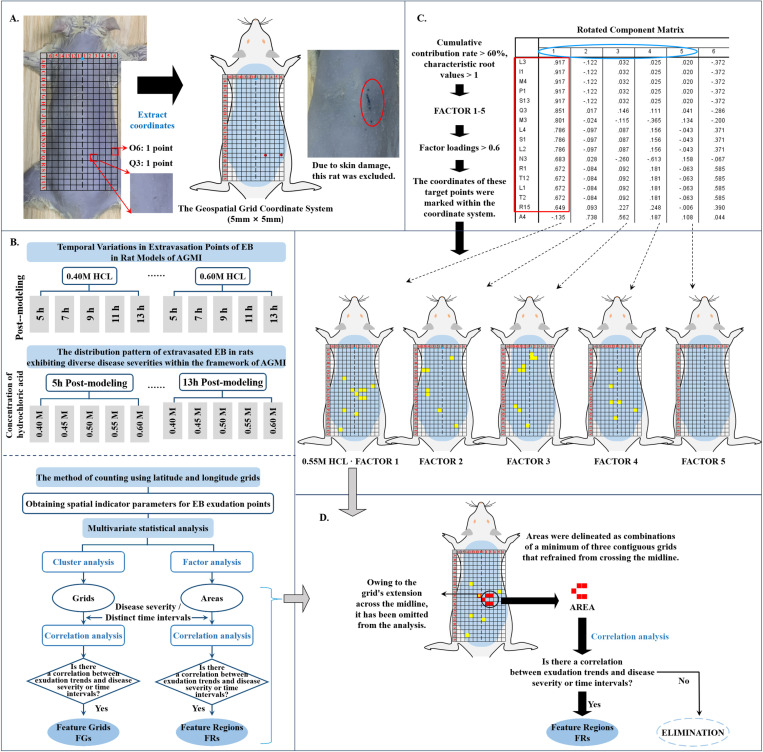
Analytical workflow for Evans blue dye extravasation in rat models of AGMI. Fig 2 presents a detailed workflow for assessing EB dye extravasation in rat models of AGMI. This comprehensive process encompasses the extraction, quantification, and spatial analysis of EB dye from the cutaneous layers of AGMI-affected rats. A. The establishment of a geospatial coordinate grid facilitates the systematic mapping of EB extravasation across the skin. Notably, rats presenting with scraping injuries were rigorously excluded from the analysis to preclude confounding effects and ensure the reliability of data related to disease-specific EB extravasation. B. A schematic overview of the methodological steps adopted for the analysis. C. A depiction of the factor analysis approach, exemplified by the group treated with 0.50 M HCl to induce AGMI. D. A diagrammatic representation of the cutaneous regions demarcated for EB dye accumulation assessment.

Induction of anesthesia in rats was achieved through the administration of 2% sodium pentobarbital, followed by the careful removal of dorsal hair to expose the corresponding back area. A transparent laminated paper bearing a 5 mm × 5 mm geospatial grid coordinate system was then placed on the rat’s back, aligning the grid’s origin with the first vertebra of the thoracic spin. The areas superior and inferior to the Feature Region (FR), as well as lateral areas, were accurately identified and marked. The dorsal BL-21 (Weishu) acupoints, situated approximately 5 mm laterally to the 13th sub-thoracic vertebra [27,28], were used as the origin to outline a grid-sized skin area on both sides of the back (Fig 2a).

### 2.5 Electroacupuncture (EA) manipulation

The methodology involved immobilizing rats using a restrainer and meticulously removing their dorsal hair to expose the back area. The EA procedure was carried out employing pairs of unipolar stainless-steel acupuncture needles (0.25 mm × 13 mm). An acupoint nerve stimulator (HNAS-200, Nanjing Jisheng Medical Technology Company, Nanjing, China) was harnessed for the experiment. The parameters for electrical stimulation included amplitude-modulated waves with a frequency of 2 Hz [29,30] and a stimulating intensity of 0.5 mA [31], with the electrical stimulation duration set at 20 minutes.

In this study, the dorsal BL-21 (Weishu) acupoints were targeted, with pairs of needles inserted perpendicularly into the regions to a depth of approximately 6 ~ 10 mm, employing a specific focal electric stimulation mode. In this mode, the positive and negative electric needles were separated by 1 mm [32,33]. For the dorsal FR, three sterile needles were vertically inserted into specially identified dorsal grids, determined via multivariate statistical analysis, also to a depth of 6–10 mm, with the first and third needles connected to the electrodes. Anatomical cross-sectional studies of Wistar rats indicate that this depth typically penetrates the dermis, hypodermis, and superficial muscle layers (e.g., latissimus dorsi muscles), although tissue layer thickness may vary slightly across dorsal regions.

The EA procedure was initiated precisely 13 hours post-modeling, carried out once per day, for a consecutive period of 7 days. During the treatment regimen, the saline (control) and model groups were retained within the fixture, devoid of any measurements or stimulations. It is essential to highlight that the rats remained conscious throughout the intervention. The consistency of the assistant and operator conducting all procedures was ensured, thereby upholding experimental protocol uniformity and standardization.

### 2.6 Rat gastric injury evaluations

Utilizing 2% pentobarbital sodium at a dosage of 50 mg/kg for anesthesia, rats underwent cardiac perfusion with normal saline, subsequent to which their stomachs were excised and immediately immersed in cold physiological saline. The specimens were then arranged on filter paper, enabling a thorough investigation of the macroscopic characteristics of gastric ulcers and erosive hemorrhagic lesions, noting their size, shape, and precise anatomical positioning. The assessment of ulcer severity adhered to the guidelines established by Guth [34]. The scoring protocol for the lesions observed was implemented in the following manner: (1) Punctured bleeding, identified as small-spot hemorrhagic erosions or gastric mucosal defects with a diameter less than 1 mm, termed punctured ulcers, was quantified such that every trio of punctured ulcers garnered 1 point. (2) For strip bleeding, vernier calipers were employed to ascertain the maximum length and width diameters of the ulcer, the latter measured perpendicular to the length. The scoring for each ulcer was derived by multiplying these dimensions, with the scoring system adjusted based on width: a width of 1 mm yielded 1 score per millimeter of length; a width of 2 mm, 2 scores per millimeter; and a width of 3 mm, 3 scores per millimeter. (3) The cumulative scores from punctured and strip bleeding evaluations constituted the ulcer index.

### 2.7 Histological examination

The excised gastric tissue was fixed in a 4% paraformaldehyde solution in preparation for paraffin embedding. Paraffin-embedded tissues were sectioned into slices of 5 μm, which were then deparaffinized in xylene and dehydrated in graded alcohol. Following this process, the sections were mounted onto glass slides. Hematoxylin and eosin (HE) staining was applied to the organ sections to facilitate visualization. The stained sections were observed and imaged at a magnification of 100x using a Nikon Eclipse CI microscope equipped with a NIKON digital sight DS-FI2 imaging system (Tokyo, Japan).

### 2.8 Enzyme-linked immunosorbent assay (ELISA)

The concentrations of TNF-α, IL-β, IL-6, IL-8, and IL-10 in serum and gastric tissue were quantified using ELISA kits procured from Boster Co., Ltd (Wuhan, CN). The measurements were performed on a microplate reader from Thermo Fisher Scientific (Massachusetts, USA), with the reader set to a wavelength of 450 nm. Simultaneously, the levels of 5-hydroxytryptamine (5-HT), substance P (SP), histamine (HA) and transient receptor potential vanilloid 1 (TRPV1) in skin tissue were determined using corresponding specific ELISA kits from the same manufacturer, with readings taken at the same wavelength.

### 2.9 Immunofluorescent staining

Rats underwent transcranial perfusion with 4 °C saline, following which skin samples were harvested. The procured tissues were then fixed in 4% paraformaldehyde at 4°C for 24 hours. Sequential dehydration was carried out in a two-step process: initially in a 20% sucrose solution for 24 hours, followed by a 30% sucrose solution for an additional 24 hours. Skin tissues were then serially sectioned into 30 μm thick slices using a frozen microtome (Thermo NX50, USA) in preparation for immunofluorescence. The sections were blocked with 5% goat serum in TBST (containing 0.3% Triton X-100) for 1 hour at 37 °C. The sections were then incubated overnight at 4 °C with primary antibodies: CALCA antibody (#DF7386, 1:200, rabbit polyclonal, Affinity, USA) and Anti-Serotonin antibody (#ab66047, 1:200, goat monoclonal, Abcam, USA). Post a 10-minute wash with 0.1 M PBS, the tissue was exposed to either Alexa Fluor 488 donkey anti-goat (ab150129, 1:200, Abcam, USA) or Alexa Fluor 594 goat anti-rabbit (ab150129, 1:200, Abcam, USA) IgG secondary antibody for 2 hours. This was followed by a wash with 0.1 M PBS. The tissue was then counterstained with DAPI nucleic acid (D3571, 1:40000, Invitrogen, USA) for 5 minutes to label the cell nuclei. Images were randomly selected from 3 sections per animal using a fluorescence microscope (DM IL LED, Leica, Germany), with uniform microscope settings maintained throughout all image capture sessions. Image analysis was conducted using ImageJ software by an investigator blinded to the experimental groups.

## 3 Data processing and analysis

### 3.1 Construction of a 5 mm × 5 mm geospatial grid coordinate system

To thoroughly investigate the complex spatial properties of the body surface, a sophisticated coordinate system analysis model was developed on the dorsal plane of the rat. Notably, the blank and saline groups, both devoid of any detectable extravasation, were exclusively employed as controls in this study. The rat’s first vertebra of the thoracic spin provided the origin of the coordinate frame, with the dorsal spine distinctly identified as the longitudinal axis. A geospatial grid coordinate system of 5 mm × 5 mm was established for each AGMI rat, based on the average 5 mm distance between each spike. The rightward horizontal axis from the origin was sequentially labelled from 1 to 6, while the leftward horizontal axis was labelled from 11 to 16. The vertical axis was denoted as A through V. As such, the extensive dorsal region of the rat was systematically divided into numerous grids, each assigned a unique identifier ranging from A1 to V16. Uncovered grids, such as A16, were not recorded. A series of rigorous steps were strictly followed to ensure precision in grid construction, including maintaining the rats’ body weight precisely at 24 hours post-feeding and a consistent camera height from the ground. Noteworthy is the use of a standard scale (1 cm) for each meticulously examined rat image, precisely aligned with the ruler scale within the images (Fig 2A).

### 3.2 Quantification of Evans blue (EB) dye extravasation points

Consistent with previous studies [23,25], the primary extravasation site of EB dye in rodents is subcutaneous, not epidermal, a critical distinction given that surface skin extravasation or intense coloration could erroneously suggest extravasation. Our findings suggest that cutaneous injuries in rodents can significantly affect disease-related extravasation on the body surface, complicating the discernment of whether injury site extravasation includes disease-associated surface extravasation. As such, it is essential to exclude such instances (Fig 2A). In this study, we meticulously documented the number of extravasated EB dye points within the A1-V16 grid for each rat. We adhered to the following principles for documentation: (1) If no discernible EB extravasation points were observed in a grid, the grid was recorded as 0. (2) If *n* intact EB extravasation points were observed within a single grid, the grid was recorded as *n.* (3) For extravasation points spanning multiple grids, specific recording principles were followed. In particular, these points were recorded within the corresponding grids, with priority given to the upper and/or right grid over the lower or left grid.

### 3.3 Multivariate statistical analysis

Statistical analyses were performed using SPSS (version 22 for Windows, SPSS Inc., USA), with continuous variables represented as mean ± standard deviation (SD). Comparisons among multiple groups were made using the Kruskal Wallis test with Dunn’s multiple comparison post-test. Multivariate statistical analysis techniques, including cluster analysis, factor analysis, and principal component analysis, were employed to comprehensively decipher the intricate interrelationships and patterns across multiple variables, providing a holistic understanding of the data structure (Fig 2B). Statistical significance was defined as a *P*-value less than 0.05.

The spatial indices of EB extravasation points were subjected to cluster analysis to identify coordinates with the most significant clustering. A subsequent correlation analysis was used to examine the relationship between extravasation, disease severity (measured by HCL concentration), and time point. In cases where a significant correlation was detected, it was classified as a feature cell (FC).

Factor analysis was applied to index parameters at each temporal point and disease severity level. The common factor was primarily identified based on Principal Component Analysis (PCA), emphasizing cumulative contribution rates exceeding 60% and characteristic root values surpassing 1 (Fig 2C). Subsequently, grid coordinates with a factor loading above 0.6 were extracted for each qualifying common factor and systematically mapped onto the corresponding rat coordinates. Combinations of at least three adjacent grids that did not cross the midline were labelled as Areas 1, Areas 2, etc., (Fig 2D). Finally, a correlation analysis was performed to explore the relationships between these defined areas, time points, and disease severity. In cases where a significant correlation was identified, the respective area was designated as a Feature Region (FR).

## 4 Results

### 4.1 Delineating the temporal and severity-dependent distribution patterns of Evans blue extravasation points in AGMI rats through multivariate analysis

#### 4.1.1 Concentration- and time-dependent morphological changes in rat gastric mucosa following hydrochloric acid exposure.

**Fig 3:**
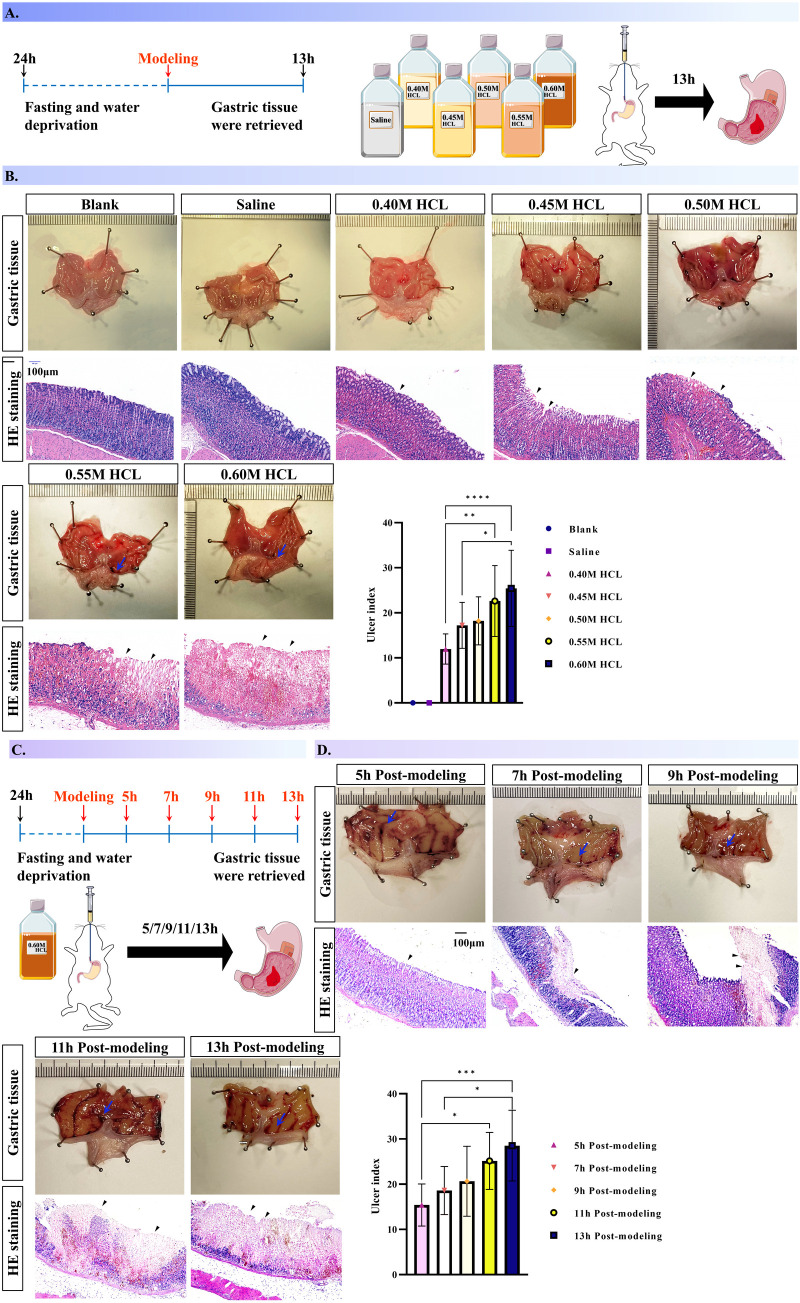
Gastric tissue damage in rats following gavage with hydrochloric acid: Effects of concentration and modeling time. Fig 3 captures the alterations in gastric tissue morphology resulting from gavage administration of hydrochloric acid (HCl) in rats, with the extent of damage modulated by both the concentration of HCl and the duration of the modeling procedure. Histopathological insights are provided through hematoxylin and eosin (H&E) staining (*n* = 6 per group). A. A schematic representation of the rat modeling protocol employed to induce gastric mucosal injury. B. Histopathological micrographs at 100 × magnification, demonstrating the concentration-specific tissue damage in the stomach, as evidenced by H&E staining, and ulcer index. C. A diagram highlighting the temporal impact of HCl exposure on gastric mucosa morphology. D. Histopathological micrographs at 100 × magnification, showcasing the time-dependent evolution of gastric tissue damage following HCl gavage, as delineated by H&E staining, and ulcer index.

To elucidate the effects of hydrochloric acid (HCl) concentration and exposure duration on the morphology of rat gastric mucosa, initially, we administered varying concentrations of HCl to AGMI rats and monitored the resultant gastric tissue alterations (Fig 3A). The control and saline-treated rats exhibited no gastric mucosal damage post a 13-hour interval, as evidenced by the absence of injury or haemorrhage. In contrast, a dose-dependent increase in gastric mucosal damage, manifested as congestion and ulceration, was noted in response to escalating HCl concentrations. Specifically, the severity of gastric lesions, quantified using the ulcer index, exhibited a significant increment at a concentration of 0.60 M HCl compared to those observed at 0.40 M and 0.45 M HCl concentrations. Furthermore, lesions at a concentration of 0.55 M HCl were markedly more severe than those at 0.45 M, with the differences reaching statistical significance (*P* < 0.001, 0.01, 0.05). Histological analysis via hematoxylin and eosin staining corroborated these findings, showing intact mucosal architecture in control and saline specimens, whereas HCl-treated rats displayed a concentration-dependent infiltration of inflammatory cells and erythrocytes within the mucosal layer, culminating in extensive damage at the highest concentrations of 0.55 M and 0.60 M HCl (Fig 3B).

Subsequent temporal assessment of gastric mucosal injury in the presence of 0.60 M HCl revealed progressive exacerbation of tissue damage over time (Fig 3C). Specifically, the ulcer index at 13 hours post-exposure was demonstrated a significant increase compared to measurements taken at 5 and 7 hours post-exposure. Additionally, a marked increase in the ulcer index was observed at 11 hours post-exposure relative to 5 hours post-exposure, with these differences achieving statistical significance (P < 0.001, 0.05). This escalation in tissue damage was accompanied by augmented infiltration of inflammatory cells, noted at sequential time points of 5, 7, 9, 11, and 13 hours post-exposure. This pattern of damage was visually and histologically documented, with diffuse congestion and ulceration becoming more pronounced over time (Fig 3D).

#### 4.1.2 Cluster analysis of temporal and severity-dependent Evans blue extravasation points in AGMI rats.

**Fig 4:**
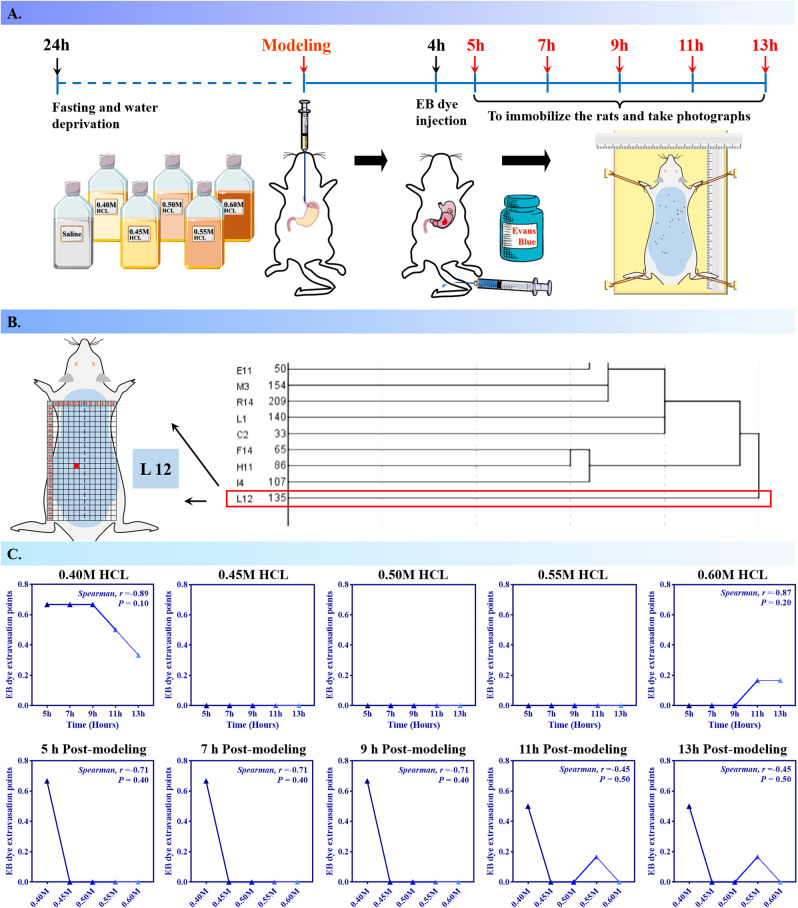
Cluster analysis of cutaneous Evans blue extravasation points in rats with AGMI. Fig 4 illustrates the cluster analysis of Evans Blue (EB) dye extravasation points on the body surface of rats with induced gastric mucosal injury, depicting the patterns of vascular leakage in relation to hydrochloric acid (HCl) concentration and exposure time (*n* = 6 per group). A. A schematic representation of the rat modeling protocol employed to induce gastric mucosal injury. B. A visual summary of the cluster analysis results, highlighting the patterns of EB dye extravasation across the rats’ body surface. C. Correlation analysis between the clustering of EB extravasation points and the variables of HCl concentration and duration of exposure.

The distribution of EB dye extravasation along the dorsal surface of AGMI rats, highlighting the effects of varying hydrochloric acid (HCl) concentrations and exposure durations on EB exudation (S1 Fig). This investigation utilized cluster analysis to meticulously examine the temporal and severity-dependent modifications in extravasation sites of EB dye in AGMI rats (*n* = 6 per group, Fig 4A). The L12 cell coordinate variable emerged as the most salient clustering in the analysis (Fig 4B). A subsequent correlation analysis was undertaken to clarify the extravasation trend concerning time and HCL concentration. Despite the absence of statistically significant correlations (*P* > 0.05), discernible patterns of EB extravasation, either decreasing or increasing, were observed over time in rats exposed to 0.40 M and 0.60 M HCL concentrations. Conversely, no evident trends were detected across different HCL concentrations (Fig 4C).

#### 4.1.3 Factor analysis of temporal and severity-dependent Evans blue extravasation points in AGMI rats.

**Fig 5:**
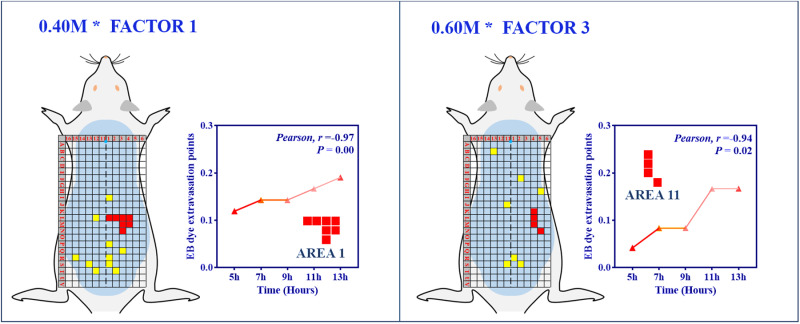
Factor analysis of temporal effects on gastric mucosal injury in rats. Fig 5 showcases a segment of the comprehensive factor analysis undertaken to decode the temporal influence on the severity of gastric mucosal injury in rat models (*n* = 6 per group).

Our investigation, through cluster analysis, revealed that both primary and secondary clusters each form a single cell. This phenomenon could potentially be attributed to the inherent limitations of assessing similarity based on frequency and distance [35], as this method often fails to capture the complex interrelationships among grids [36]. To circumvent this, we employed factor analysis as an additional tool, implemented independently across disease severity and temporal dimensions, with the primary aim of identifying potential combinations of extravasation sites exhibiting shared expression patterns. Within the framework of disease severity, significant correlations were identified between AREA 1 and AREA 11, and temporal alteration (0.40 M, *Spearman*, *r* = 0.97, *P* < 0.00) (0.60 M, *Pearson*, *r* = 0.94, *P* < 0.05, Fig 5). These areas were consequently designated as Feature Regions (FRs) in our study, specifically FR-1 and FR-11. Despite not all correlations achieving statistical significance, certain areas exhibited an extravasation trend in association with temporal shifts. However, within the temporal dimension, it is crucial to underscore the lack of statistically significant correlations between areas and modifications in HCL concentration (S2 Fig).

### 4.2 Regional discrepancies in the expression of neuroinflammatory mediators on the body surface of AGMI rats

**Fig 6:**
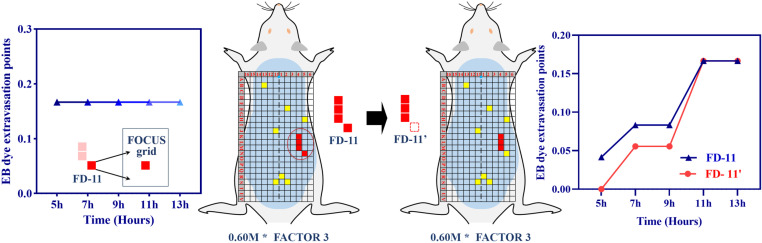
Repeated measures variance analysis of feature regions 11 and 11’. Fig 6 delineates the outcomes of a repeated measures analysis of variance (ANOVA) performed on Feature Regions 11 (FR-11) and 11’ (FR-11’).

In the present investigation, we further clarified the specific expression of neuroinflammatory mediators in the skin tissues of Feature Regions (FRs). The designation FR-1 refers to the results obtained from a 0.40 M HCL concentration analysis. Given the remarkable self-healing capability exhibited by rats, we aimed to validate our findings by selecting FR-11, which was derived from rats exposed to a 0.60 M HCL concentration and encompasses grids K4, L4, M4, and N5. Temporal consistency analyses revealed a lack of association for grid N5 (*P* > 0.05), prompting its exclusion and the subsequent establishment of FR-11’, now comprising K4, L4, and M4. To explore potential disparities in extravasation trends pre and post this regional geometric redefinition, we conducted a repeated measures ANOVA. The analysis failed to detect significant differences in extravasation trends between FR-11 and FR-11’ (*F* = 0.32, *P* = 0.859), suggesting comparable levels across both regions at different time points. Based on these findings, FR-11’ was selected for comprehensive subsequent analysis (Fig 6).

**Fig 7:**
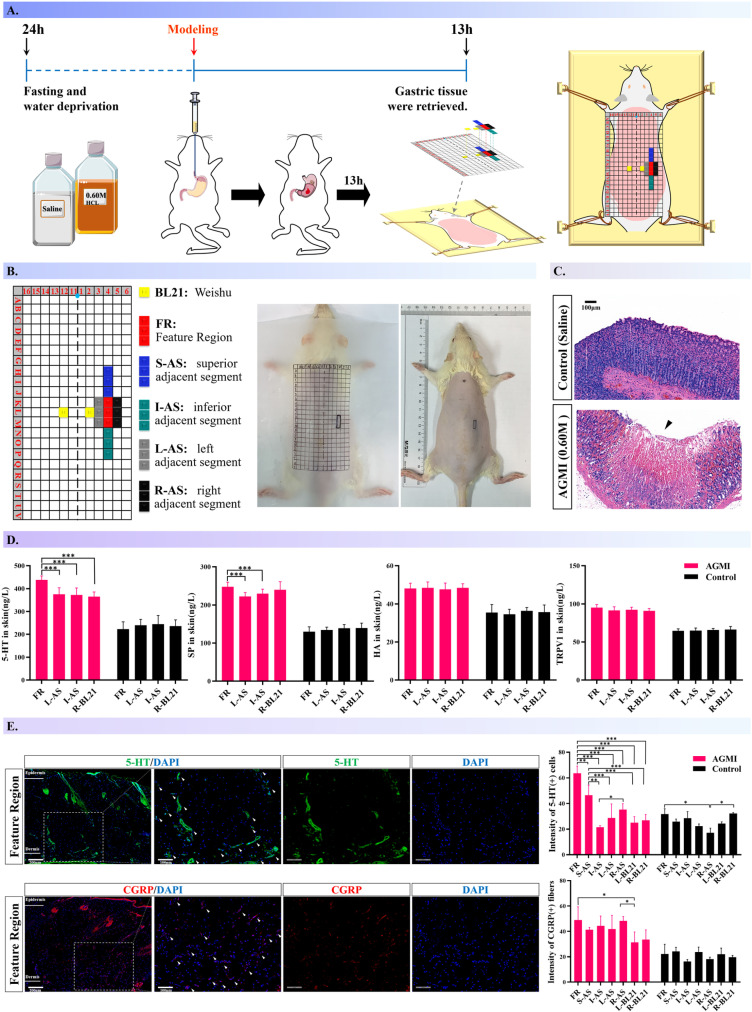
Mapping neuroinflammatory responses on rat dorsal surfaces post-AGMI. Fig 7 synthesizes the investigation of neuroinflammatory mediator expression across a range of dorsal cutaneous regions in rats subjected to AGMI. A. Schematic representation of the methodological approach employed to investigate neuroinflammatory responses. B. Schematic localization of the multiple dorsal body surface areas assessed for neuroinflammatory activity in the rat model. C. Histological images of gastric tissue from both AGMI-induced and control rats, stained with Hematoxylin and Eosin (H&E) at 100x magnification. D. Comparative evaluation of nociceptive neuropeptides and sensitizers in predefined cutaneous regions between control groups and AGMI-treated rats (*n* = 6 per group). E. Immunofluorescent localization of 5-HT and CGRP in the cutaneous FR of rats with AGMI (*n* = 3 per group).

We further performed a comparative quantitative analysis of epidermal nociceptive neuropeptides— 5-HT and SP—alongside HA and TRPV1, focusing on the FRs in AGMI rats relative to control animals treated with saline, as well as to adjacent areas and established acupoints (Fig 7A-B). We first confirmed the successful establishment of the AGMI model using HE staining (Fig 7C, S7A Fig). Histological examination of intestinal mucosal damage was conducted on the duodenum, jejunum, ileum, and colon (S8 Fig). Subsequently, ELISA analyses (Fig 7D) indicate a significant elevation of 5-HT and SP levels in the FR relative to adjacent control areas (*P* < 0.001). Furthermore, 5-HT concentrations in the FR notably exceed those measured at the right-side BL-21 acupoint. In contrast, histamine and TRPV1 levels did not differ significantly between the groups (*P* > 0.05).

Subsequent immunofluorescence assays elucidated the spatial distribution of 5-HT and CGRP across a broader spectrum of cutaneous sites. The FR displayed a substantial increase in 5-HT-positive neural fibers, outstripping the adjacent areas and acupoints. Additionally, areas superior to the FR (S-AS) showed a significant rise in 5-HT levels in comparison to other skin sites (*P *< 0.001). CGRP distribution was notably higher within the FR and the immediate right-side regions (R-AS) than in the left BL-21 acupoints (*P *< 0.05). For an in-depth examination of the immunofluorescence results pertaining to the FR in rats subjected to AGMI, refer to Fig 7E. Detailed visual representations are provided in S3-S6 Figs, offering an extensive overview of the findings.

### 4.3 Assessing electroacupuncture (EA) efficacy at feature regions (FR) in AGMI rats

**Fig 8:**
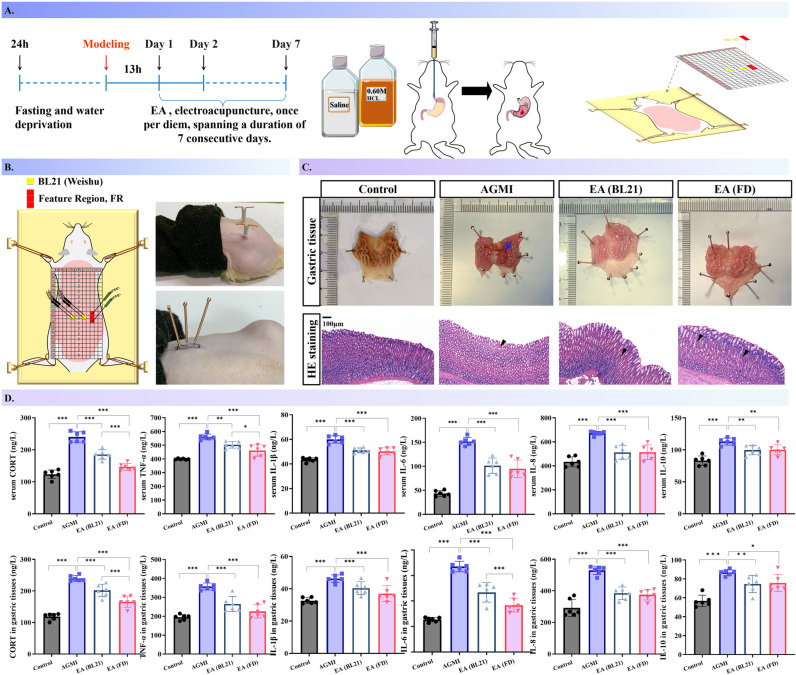
Influence of Electroacupuncture at Feature Regions on Gastric Mucosal Healing in AGMI Rats. Fig 8 examines the therapeutic efficacy of electroacupuncture (EA) applied at feature regions (FR) on healing gastric mucosal lesions in a rat model of AGMI (*n* = 6 per group). A. A schematic representation of the rat modeling protocol employed to induce gastric mucosal injury. B. A diagram indicating the exact locations of EA intervention on the rats’ body. C. Histological sections of the gastric mucosa after a seven-day course of EA treatment, stained with Hematoxylin and Eosin (H&E) and presented at 100x magnification. D. A comparative evaluation of cytokine concentrations in both serum and gastric tissue samples.

To assess the potential therapeutic efficacy of the identified FR, AGMI was induced in rat models via gavage administration of 0.60 M HCL. This was followed by the application of EA as an intervention strategy (*n* = 6 per group, Fig 8A-B). Macroscopic examination of the gastric tissue after a seven-day intervention period revealed well-defined, undamaged gastric folds in both the saline and EA-treated groups. In contrast, the AGMI group displayed gastric tissue with sporadic congestion and ulceration. Histological analysis of HE-stained gastric tissue demonstrated that the gastric mucosal epithelium in saline-treated rats remained intact, with no signs of inflammatory cell infiltration. However, the AGMI group exhibited compromised gastric mucosal epithelium, lax tissue structure, inflammatory cell infiltration, and occasional presence of erythrocytes within the tissue. In the EA-treated groups, the gastric mucosal epithelium structures largely maintained normalcy, with a tendency towards regular glandular arrangement, slightly lax structure, and no obvious inflammatory cell infiltration or congestion (Fig 8C, S7B Fig).

Furthermore, ELISA data revealed significantly increased levels of inflammatory cytokines in both serum and gastric tissue of AGMI group rats compared to saline-treated controls (*P* < 0.001). In contrast, the corresponding indicators in the EA groups were significantly subdued compared to the AGMI group (*P* < 0.001, *P* < 0.01, *P* < 0.05). Of particular note, the EA-FD group manifested significantly diminished levels of cortisol (CORT) in both serum and gastric tissue, as well as TNF-α in serum and IL-6 in gastric tissue, compared to the EA-BL21 group (*P* < 0.001, *P* < 0.05, Fig 8D).

## 5 Discussion

**Fig 9:**
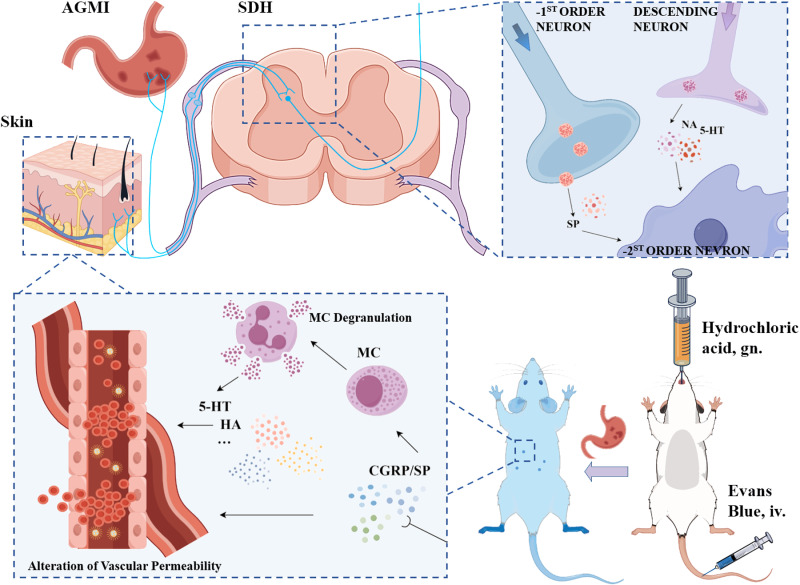
Potential mechanistic pathways of neurogenic skin responses post-visceral sensitization following AGMI. Fig 9 outlines a conceptual framework illustrating the mechanistic pathways involved in the development of neurogenic skin responses following visceral sensitization subsequent to AGMI. In pathological conditions, abnormal excitatory signals originating from the viscera are transmitted via dorsal root neurons, subsequently activating spinal dorsal horn cells and propagating to peripheral nerve terminals. Upon entering the spinal dorsal horn, these nerve impulses activate additional dorsal root ganglion cells through interneurons and are then transmitted to peripheral nerves via dorsal root reflexes. Furthermore, abnormal excitatory signals from diseased viscera are conveyed to peripheral nerves through somatovisceral reflexes via dorsal root branches. Upon reaching the periphery, these impulses stimulate nerve terminals to release inflammatory mediators such as SP and CGRP, thereby eliciting a neurogenic inflammatory response. Subsequently, SP further induces MC aggregation and degranulation, leading to the release of algogenic substances including 5-HT and HA, which result in localized cutaneous hyperalgesia. Labels: CGRP: Calcitonin gene-related peptide; SP: Substance P; MC: Mast cell; 5-HT: 5-Hydroxytryptamine hydrochloride; HA: Histamine; NA: Noradrenaline; AGMI: Acute gastric mucosal injury; SDH: Spinal dorsal horn; i.g.: Oral gavage; i.v.: Intravenous injection.

Our study reveals that AGMI induces neurogenic plasma extravasation primarily within the right T10-13 dermatomere (FR-11’, a grid combination of K4, L4, and M4) in the skin, exhibiting a line segment pattern parallel to the spine. A temporal correlation with increasing extravasation was observed over a 13-hour post-mold creation period. The distribution of nociceptive neuropeptides (CGRP and SP) and allergic substances (5-HT) was significantly higher in FR-11’ compared to adjacent regions and/or the traditional acupoint (BL-21). EA at the FR demonstrated superior efficacy, as evidenced by significantly reduced cortisol and certain inflammatory markers in both serum and gastric tissue, compared to BL-21 (Fig 9).

EB dye, widely recognized for its utility in assessing tissue vitality in both clinical and mechanistic research [37–40], binds selectively to serum albumin to form a stable, high molecular weight complex that remains confined to the circulatory system [41,42]. Its unique property of labeling necrotic tissue without affecting healthy cells or breaching the blood-brain barrier has positioned it as a valuable tool in biomedical studies [43]. Emerging evidence links EB extravasation in the skin to neurogenic inflammation induced by visceral disorders [2,8], where the convergence of viscerosomatic sensory pathways manifests as cutaneous inflammation. This suggests that local allergic mediators and nociceptive neuropeptides may play a role in the sensitization process [14,19,44]. However, these were isolated points, and seldom did two rats share the same exudation point, including location and number. Therefore, when endeavoring to study this surface nerve inflammation, the initial challenge is to determine which points are disease-related.

To enhance the precision of localizing EB extravasation points, we utilized spatial autocorrelation analysis, a technique pioneered by Cliff et al. in 1973, which incorporates multivariate statistical methods such as spatial clustering and factor analysis [45,46]. This approach involves calculating spatial autocorrelation coefficients for a given spatial unit in relation to its adjacent units, thereby facilitating a detailed examination of their distribution within the spatial domain [47,48]. By conceptualizing EB extravasation points as geographical markers on the body surface, we developed a geospatial grid coordinate system, assigning exact coordinates to identify the EB extravasation sites. This methodology allowed us to establish statistical relationships based on spatial positioning. Moreover, taking into account the spacing of the vertebras and the actual surface area of the rats’ bodies, we adopted a measurement unit of 5 mm × 5 mm, enabling a comprehensive analysis of the distribution patterns observed.

We extended the use of multivariate statistical analysis to examine the EB grid coordinates. Initial cluster analysis suggested the highest degree of clustering at the L12 cell coordinate, yet the identification of disease-associated variations proved challenging, likely due to intrinsic constraints of the cluster analysis technique [49,50]. This method, traditionally focused on grouping objects based on frequency and distance, often neglects the complex interplay between grid coordinates, impeding the detection of nuanced disease-related patterns [36].

To uncover the complex interconnections among variables, our study employed factor analysis, a method esteemed for its efficacy in dimensionality reduction and variable selection [51,52]. It excels in distilling essential information from a vast dataset and constructing latent factor models that reveal underlying data structures [53,54]. Factor analysis discerns latent factors that encapsulate common dimensions accounting for data correlations [52], while factor loadings indicate the magnitude and direction of associations between observed variables and these latent factors, akin to correlations [55,56]. We delineated common factors with cumulative contribution rates above 60% and eigenvalues over 1, extracting grid coordinates with factor loadings greater than 0.6 to define each significant common factor.

Factor and correlation analyses identified Area-1 and Area-11 as significant within the disease severity framework, exhibiting a time-correlated EB extravasation trend, thus defining the Feature Region (FR). Area-1 corresponded to 0.40 M HCL and Area-11 to 0.60 M HCL conditions. This analysis underscores the ability of FR, as deduced through factor analysis, to elucidate the relationship between EB extravasation and temporal dynamics. The observed phenomenon, wherein FR was only detected in the lowest (FR-1, 0.40 M HCl) and highest (FR-11′, 0.60 M HCl) dose groups, maybe attributed to the threshold and maximal responses these concentrations represent. Intermediate concentrations (0.45–0.55 M) likely induce transient or diffuse neurogenic inflammation, which is insufficient to form spatially clustered FRs. This biphasic pattern reflects the dose-dependent activation of nociceptors: subthreshold doses fail to initiate sustained signaling, while supra-threshold doses overwhelm compensatory mechanisms, thereby stabilizing FR formation. Similar dose-response dynamics have been documented in neurogenic inflammation models [57], supporting our observations.

However, within the temporal level framework, no discernible area was observed at the 5-hour mark post-modelling under any condition, suggesting that regional manifestations of body surface extravasation may not occur during the disease’s initial stages. Furthermore, no areas showed significant associations with disease severity at other time points. These observations suggest variability in neurogenic plasma extravasation across disease severities, which may stabilize as the disease progresses, as evidenced by trends in Area-16 under specific conditions.

Guided by the results of preliminary studies and with the objective of refining our experimental framework, we redefined FR-1 by excluding the N5 site, yielding an updated region, FR-11’, comprising K4, L4, and M4. Repeated measures ANOVA confirmed the consistency of extravasation patterns within this morphologically altered region, with no significant differences from the original region, suggesting that the modification did not alter the fundamental extravasation dynamics over the evaluated time intervals.

Furthermore, this study provides augmented evidence supporting the localization of body surfaces. Our aim was to delineate the exact expression of neuroinflammatory mediators in the cutaneous tissues of FR. It is now understood that nociceptive sensory signals from visceral dysfunctions trigger the secretion of various neurotransmitters and neuropeptides in the dorsal horn neurons of the spinal cord, encompassing CGRP and SP [58–61]. These molecules possess the potential to modulate the secretion of local neuroinflammatory agents, including histamine, prostaglandins, 5-HT, interleukins, and more, thereby facilitating vasodilation and enhancing local blood circulation, culminating in neurogenic inflammation [57,62]. Elevated levels of such mediators, including 5-HT and SP, were detected in specific dorsal skin areas of AGMI rats, further substantiating prior findings. In AGMI rats, FR-11’ showed significantly higher concentrations of 5-HT and CGRP compared to adjacent tissues and the acupoint BL-21, emphasizing the relevance of these regions in neurogenic inflammation and visceral pain research.

It may be reasonably posited that activation of these sensitive regions underlies the therapeutic effects. The pro-inflammatory cytokine TNF-α, known for its role in initiating inflammatory cascades, and IL-6, which acts as an amplification signal [63–65], are recognized as critical mediators of inflammation progression [66–68]. Consequently, cortisol (CORT) is acknowledged for its regulatory influence on metabolic, immune, and stress response pathways [69]. Inflammatory mediators such as TNF-α and IL-6, released following gastric mucosal injury, prompt the adrenal cortex to secrete CORT, further propagating the inflammatory response [69,70].

Encouragingly, a growing body of evidence suggests that electroacupuncture (EA) can modulate inflammatory and immune responses, attenuate stress reactions, diminish sympathetic adrenal medulla axis excitability, and thereby reduce CORT secretion [71–73]. Based on these insights, we embarked on a final phase of efficacy validation through EA intervention, noting a pronounced elevation of inflammatory cytokines in AGMI rats, which was significantly mitigated by EA intervention, underscoring its therapeutic potential in dampening inflammation and promoting gastric mucosal repair. Interestingly, our findings also demonstrate that TNF-α, IL-6, and CORT are particularly sensitive indicators during inflammation, with the EA-FD group exhibiting markedly lower levels of these cytokines compared to the EA-BL21 group, highlighting the superior interventional efficacy of targeting the Feature Region (FR) over the traditional acupoint BL-21.

## 6 Limitations

Despite the auspicious implications of the results obtained in this investigation, it is essential to recognize certain constraints that warrant further scrutiny. The primary limitation is derived from the relatively brief observational period for EB extravasation, which hampers a thorough interpretation of the findings. Furthermore, repeated measures ANOVA was employed to delineate the extravasation characteristics pre- and post-geometric morphological alterations of FR-11 and FR-11’. This approach revealed a consistent trend in intensity, as validated mathematically. Nonetheless, additional biological-level research is imperative to ascertain the full magnitude of this impact. A third constraint emerges from the specific mechanism that underpins the relationship between the acquired region and visceral pain sensitization, such as the effect on dorsal root ganglia, thus necessitating further exploration.

## 7 Conclusions

In this study, we explore the intricate interplay between body-surface information and visceral sensitization, with a focus on neurogenic spots - products of cutaneous neurogenic inflammation that align with visceral afferent innervations. Despite prior research, a full understanding of these phenomena remains elusive, often hindered by quantitative limitations and uncertainty. To address this, we instigated neurogenic plasma extravasation within the skin by inducing AGMI using hydrochloric acid. Utilizing a geospatial grid system alongside multivariate analysis, we pinpointed specific grid combinations (K4, L4, M4) within the right T10-13 dermatomere, which displayed a distinct pattern parallel to the spinal column. This area, termed FR-11’, demonstrated elevated levels of nociceptive neuropeptides and allergic mediators, surpassing those in adjacent regions and the traditional acupoint BL-21. Crucially, electroacupuncture at FR-11’ exhibited enhanced efficacy in reducing inflammatory responses, highlighting its potential as an advanced target for pain management in AGMI. This investigation thus represents a significant advance in elucidating the relationship between surface neurogenic markers and visceral pain sensitization.

## Supporting information

S1 Fig Dorsal distribution of Evans Blue (EB) dye extravasation in AGMI rats. The figure illustrates the distribution of EB dye extravasation along the dorsal surface of AGMI rats, highlighting the effects of varying hydrochloric acid (HCl) concentrations and exposure durations on EB exudation (*n* = 6 per group).
(TIF)

S2 Fig Factor and Correlation Analysis of HCl Concentration and Exposure Duration on EB Extravasation in AGMI Rats. S2 Fig illustrates the results of factor and correlation analyses investigating the effects of two key variables—hydrochloric acid (HCl) concentration and exposure duration—on the distribution of EB dye extravasation points along the dorsal surface of rats with AGMI (*n* = 6 per group).
(TIF)

S3 Fig Serotonin (5-HT) Distribution in AGMI Rat Cutaneous Regions as Revealed by Immunofluorescence. S3 Fig provides a visual representation of 5-HT distribution in the skin of rats following AGMI, utilizing immunofluorescence to highlight the regional expression levels (*n* = 3 per group). This figure offers a quantitative analysis of 5-HT fluorescence intensities across various selected skin areas. Sequential panels display each targeted cutaneous region at 10x magnification, with corresponding insets showcasing enhanced detail at 20x magnification, allowing for a comparative assessment of 5-HT accumulation in the context of AGMI.
(TIF)

S4 Fig Serotonin (5-HT) Immunofluorescence in Cutaneous Tissue of Saline-Lavaged Control Rats. S4 Fig captures the immunofluorescent distribution of 5-HT in the skin tissue of control rats that underwent gastric lavage with a physiological saline solution (*n* = 3 per group). The figure comprises a series of panels, each illustrating a different cutaneous area at 10x magnification, with insets offering a more detailed visualization at 20x magnification, to document the 5-HT landscape in the absence of gastric mucosal injury.
(TIF)

S5 Fig Calcitonin Gene-Related Peptide (CGRP) Distribution in AGMI Rat Cutaneous Regions as Revealed by Immunofluorescence. S5 Fig highlights the immunofluorescent localization of CGRP within the skin of rats subjected to AGMI via HCl lavage (*n* = 3 per group). The figure provides a visual quantification of CGRP presence, with each panel focusing on a distinct cutaneous region captured at 10x magnification. Accompanying high-resolution images at 20x magnification offer detailed views of the peptide’s distribution.
(TIF)

S6 Fig Cutaneous Calcitonin Gene-Related Peptide (CGRP) Immunofluorescence in Cutaneous Tissue of Saline-Lavaged Control Rats. S6 Fig presents the immunofluorescent profiling of CGRP in the skin of control rats following gastric lavage with physiological saline (*n* = 3 per group). The figure comprises a series of panels, each illustrating a different cutaneous area at 10x magnification, with insets offering a more detailed visualization at 20x magnification, to document the CGRP landscape in the absence of gastric mucosal injury.
(TIF)

S7 Fig The Results of Ulcer Index. In the context of elucidating regional discrepancies in the expression of neuroinflammatory mediators across the body surface, S7A Fig presents the ulcer index outcomes. Notably, lesions induced by a 0.60 M HCl concentration exhibited significantly increased severity compared to those in the control group (saline), with statistical analyses confirming the significance of these differences (*P* < 0.001, *n* = 6 per group). Furthermore, S7B Fig delves into the evaluation of the impact of electroacupuncture (EA) at specific feature regions. Within this study framework, the ulcer index in the AGMI group, subjected to 0.60 M HCl, showed a pronounced elevation when contrasted with both the control group and groups receiving EA at BL21 and FD points. These variations were statistically significant (*P* < 0.001, *n* = 6 per group).
(TIF)

S8 Fig Histological examination of intestinal mucosal damage. The duodenum, jejunum, ileum, and colon were inspected 13 hours post-modeling. Notably, the mucosal injury in the intestines appeared minimal following the modeling procedure (0.60 M).
(TIF)
