# Supplementary figures and images for: Localization and characterization of cutaneous neurogenic inflammation in acute gastric mucosal injury in rats: A possible morphological explanation for visceral sensitization?
Source: PLoS One. 2025 Jun 4;20(6):e0324136. doi: 10.1371/journal.pone.0324136 (PMC12136448; doi:10.1371/journal.pone.0324136)

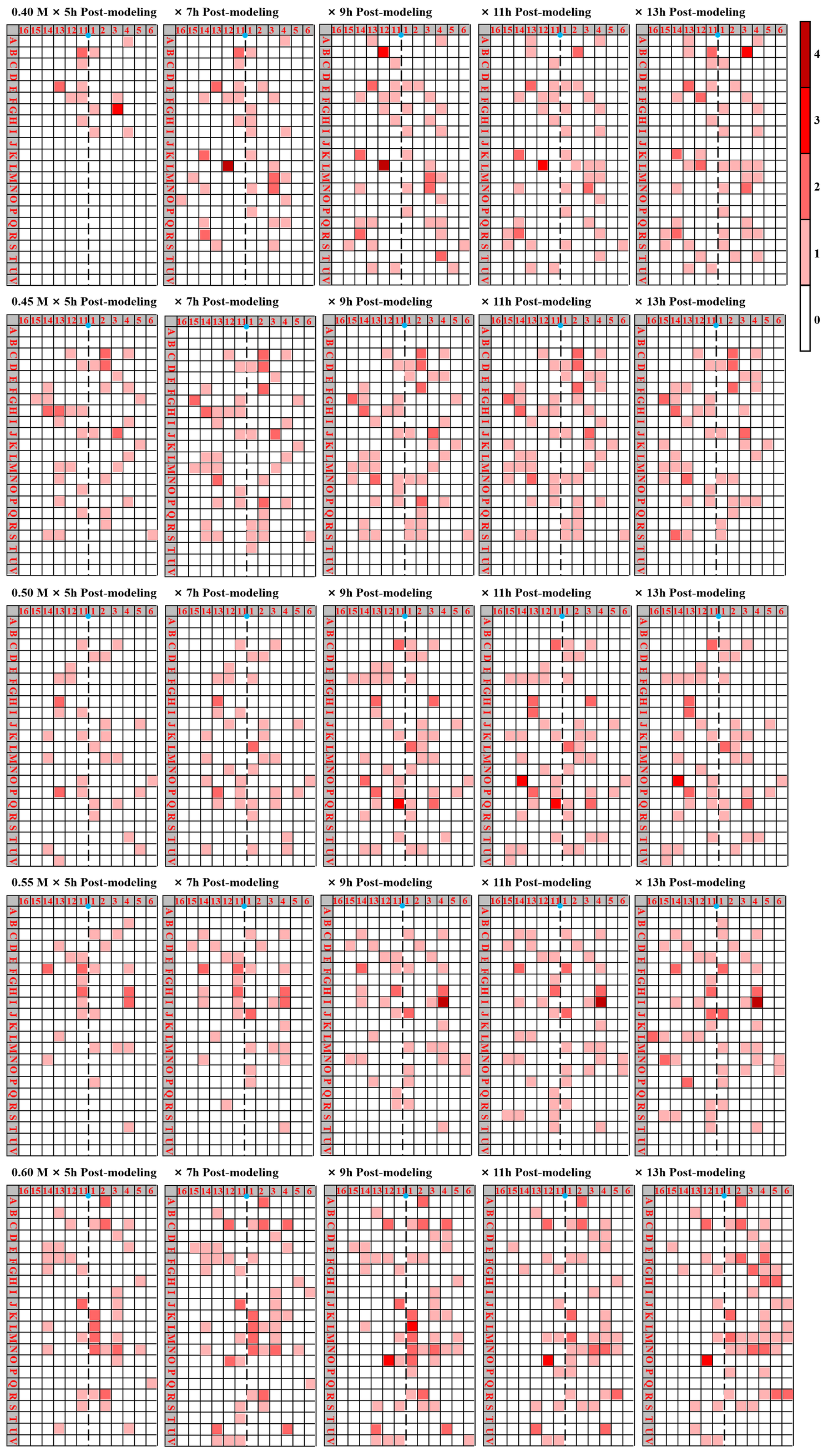

Supplement: S1 Fig — The figure illustrates the distribution of EB dye extravasation along the dorsal surface of AGMI rats, highlighting the effects of varying hydrochloric acid (HCl) concentrations and exposure durations on EB exudation (n = 6 per group). (TIF) [file pone.0324136.s001.tif]

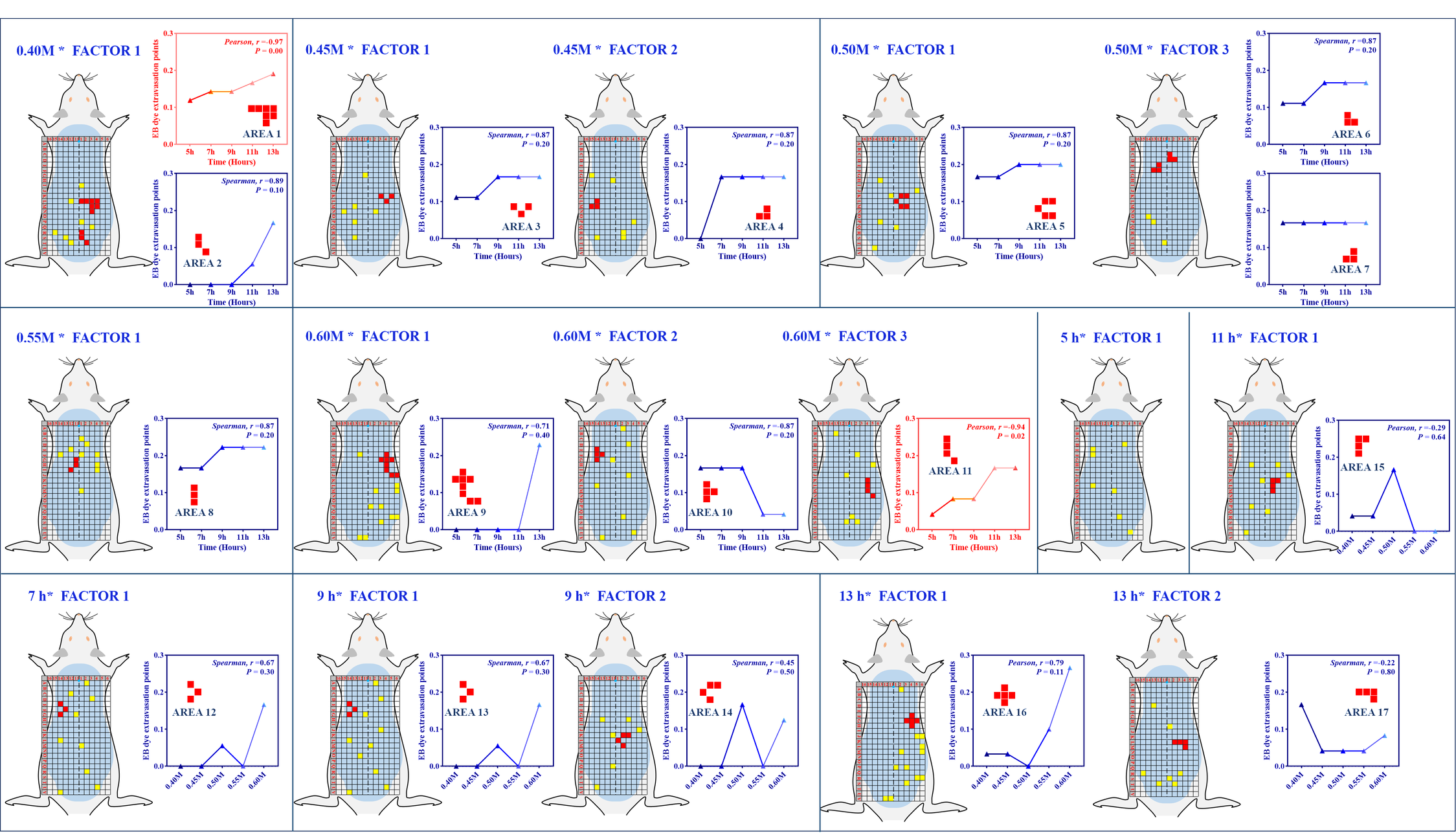

Supplement: S2 Fig — illustrates the results of factor and correlation analyses investigating the effects of two key variables—hydrochloric acid (HCl) concentration and exposure duration—on the distribution of EB dye extravasation points along the dorsal surface of rats with AGMI (n = 6 per group). (TIF) [file pone.0324136.s002.tif]

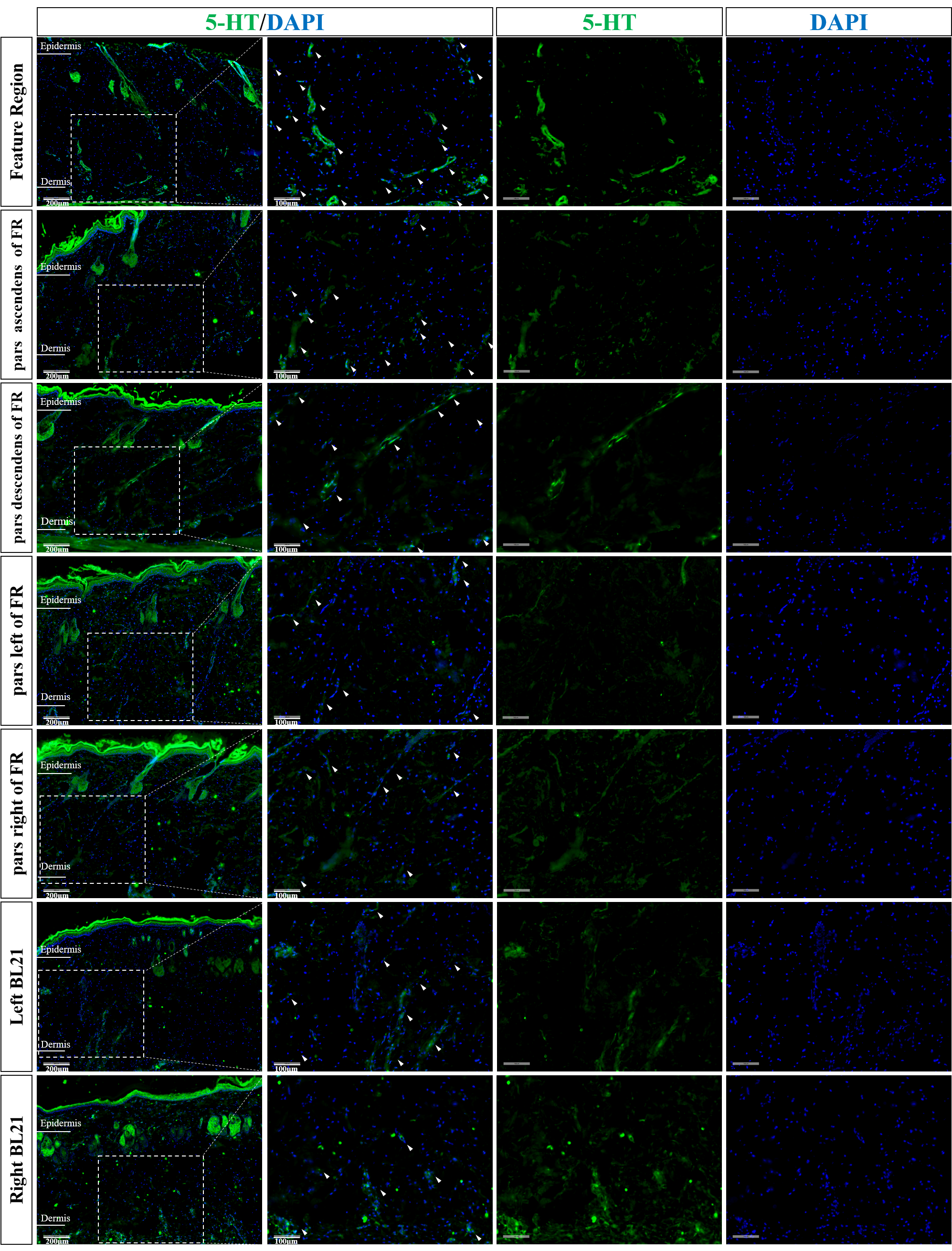

Supplement: S3 Fig — provides a visual representation of 5-HT distribution in the skin of rats following AGMI, utilizing immunofluorescence to highlight the regional expression levels (n = 3 per group). This figure offers a quantitative analysis of 5-HT fluorescence intensities across various selected skin areas. Sequential panels display each targeted cutaneous region at 10x magnification, with corresponding insets showcasing enhanced detail at 20x magnification, allowing for a comparative assessment of 5-HT accumulation in the context of AGMI. (TIF) [file pone.0324136.s003.tif]

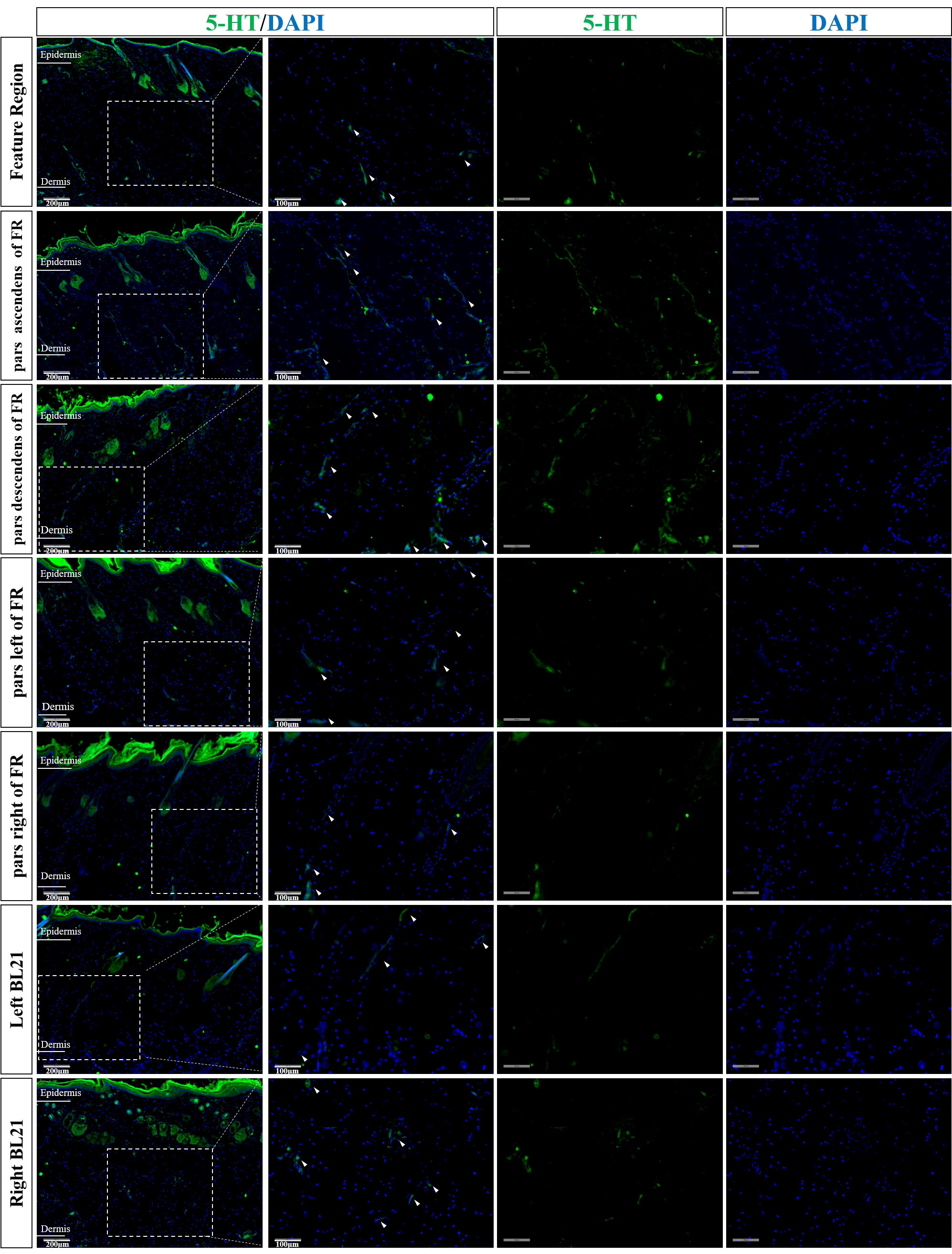

Supplement: S4 Fig — captures the immunofluorescent distribution of 5-HT in the skin tissue of control rats that underwent gastric lavage with a physiological saline solution (n = 3 per group). The figure comprises a series of panels, each illustrating a different cutaneous area at 10x magnification, with insets offering a more detailed visualization at 20x magnification, to document the 5-HT landscape in the absence of gastric mucosal injury. (TIF) [file pone.0324136.s004.tif]

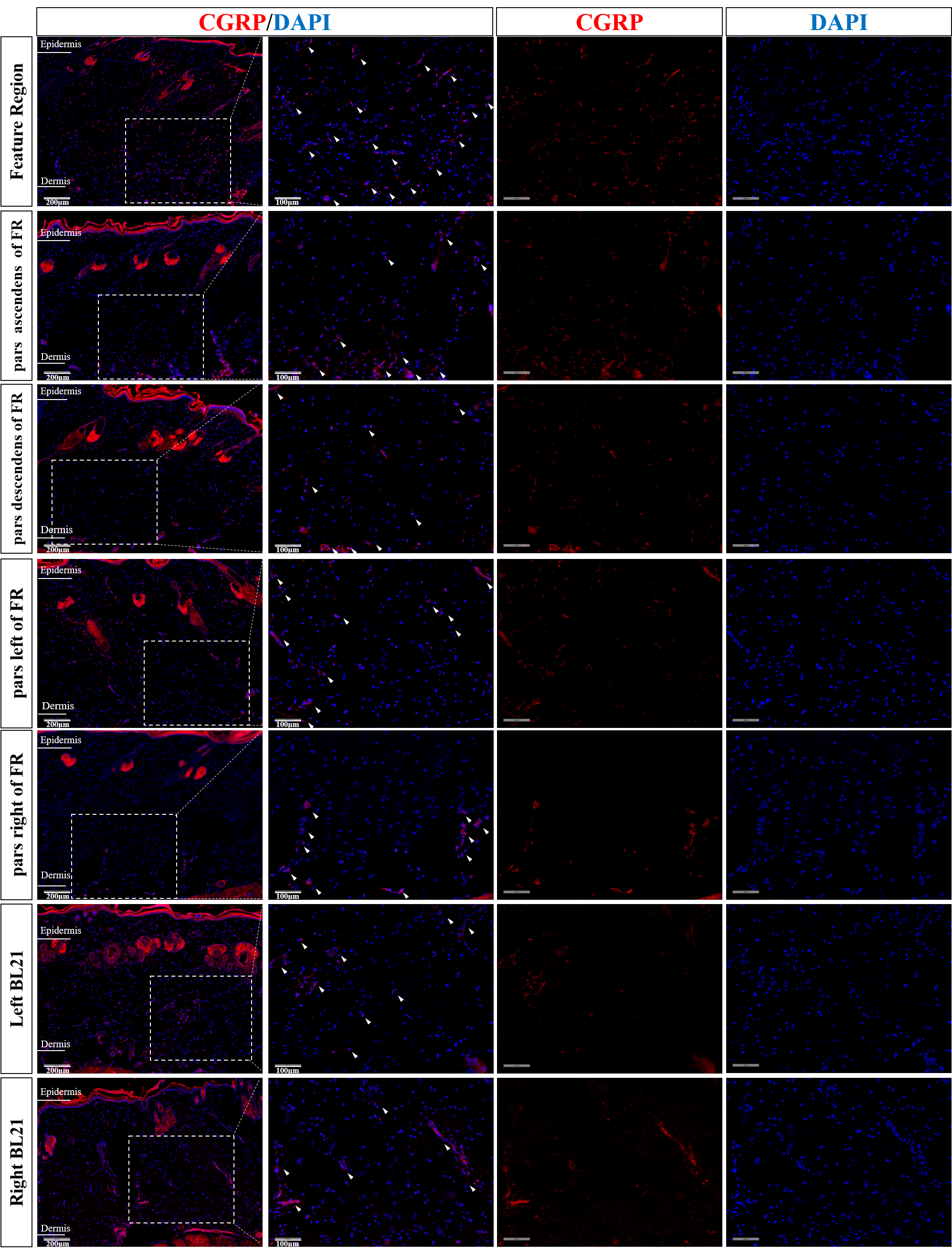

Supplement: S5 Fig — highlights the immunofluorescent localization of CGRP within the skin of rats subjected to AGMI via HCl lavage (n = 3 per group). The figure provides a visual quantification of CGRP presence, with each panel focusing on a distinct cutaneous region captured at 10x magnification. Accompanying high-resolution images at 20x magnification offer detailed views of the peptide’s distribution. (TIF) [file pone.0324136.s005.tif]

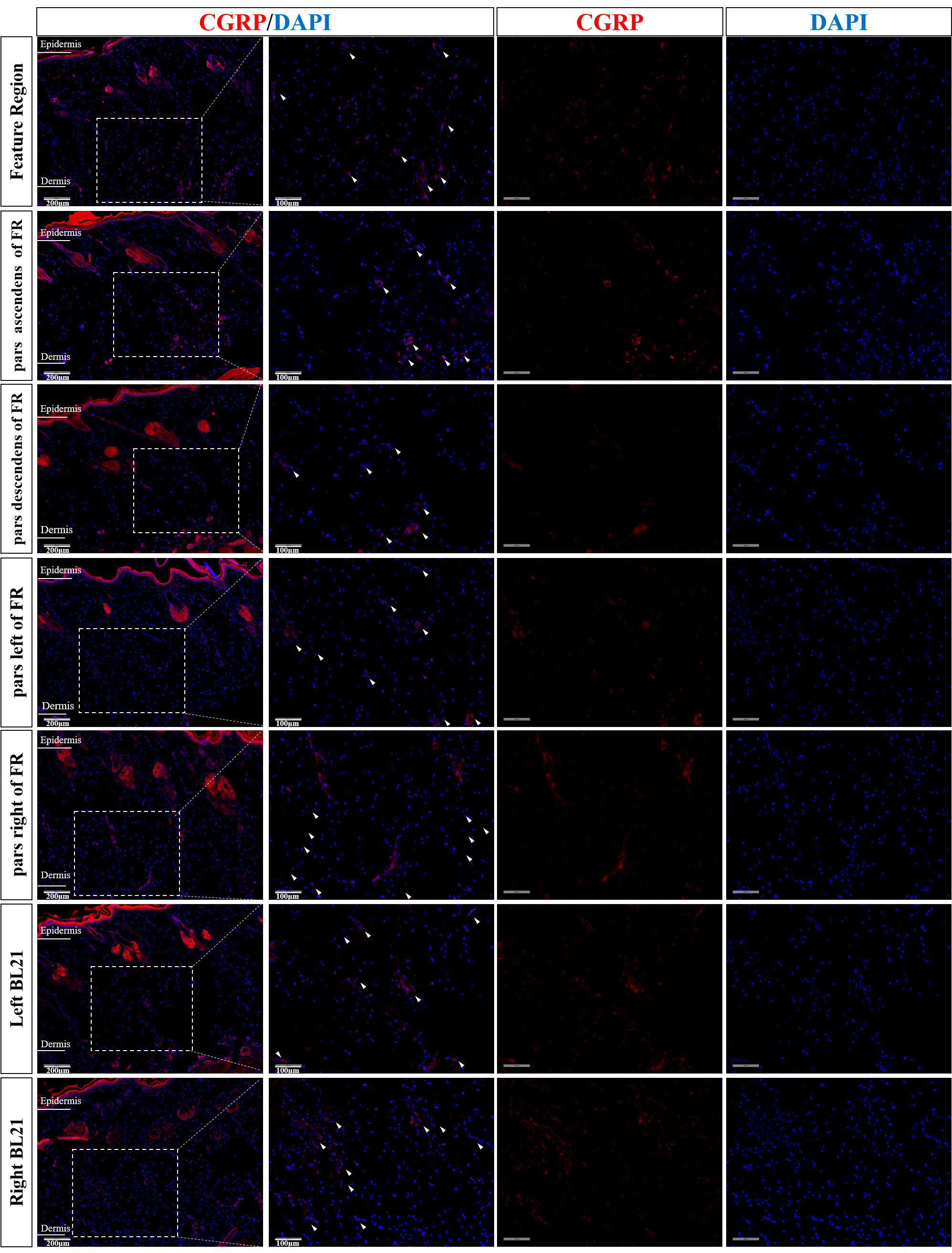

Supplement: S6 Fig — presents the immunofluorescent profiling of CGRP in the skin of control rats following gastric lavage with physiological saline (n = 3 per group). The figure comprises a series of panels, each illustrating a different cutaneous area at 10x magnification, with insets offering a more detailed visualization at 20x magnification, to document the CGRP landscape in the absence of gastric mucosal injury. (TIF) [file pone.0324136.s006.tif]

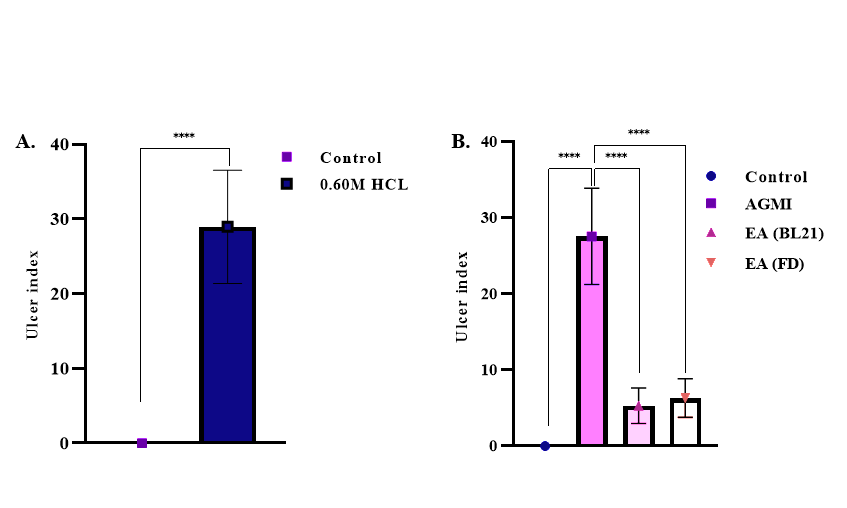

Supplement: S7 Fig — In the context of elucidating regional discrepancies in the expression of neuroinflammatory mediators across the body surface, S7A Fig presents the ulcer index outcomes. Notably, lesions induced by a 0.60 M HCl concentration exhibited significantly increased severity compared to those in the control group (saline), with statistical analyses confirming the significance of these differences (P < 0.001, n = 6 per group). Furthermore, S7B Fig delves into the evaluation of the impact of electroacupuncture (EA) at specific feature regions. Within this study framework, the ulcer index in the AGMI group, subjected to 0.60 M HCl, showed a pronounced elevation when contrasted with both the control group and groups receiving EA at BL21 and FD points. These variations were statistically significant (P < 0.001, n = 6 per group). (TIF) [file pone.0324136.s007.tif]

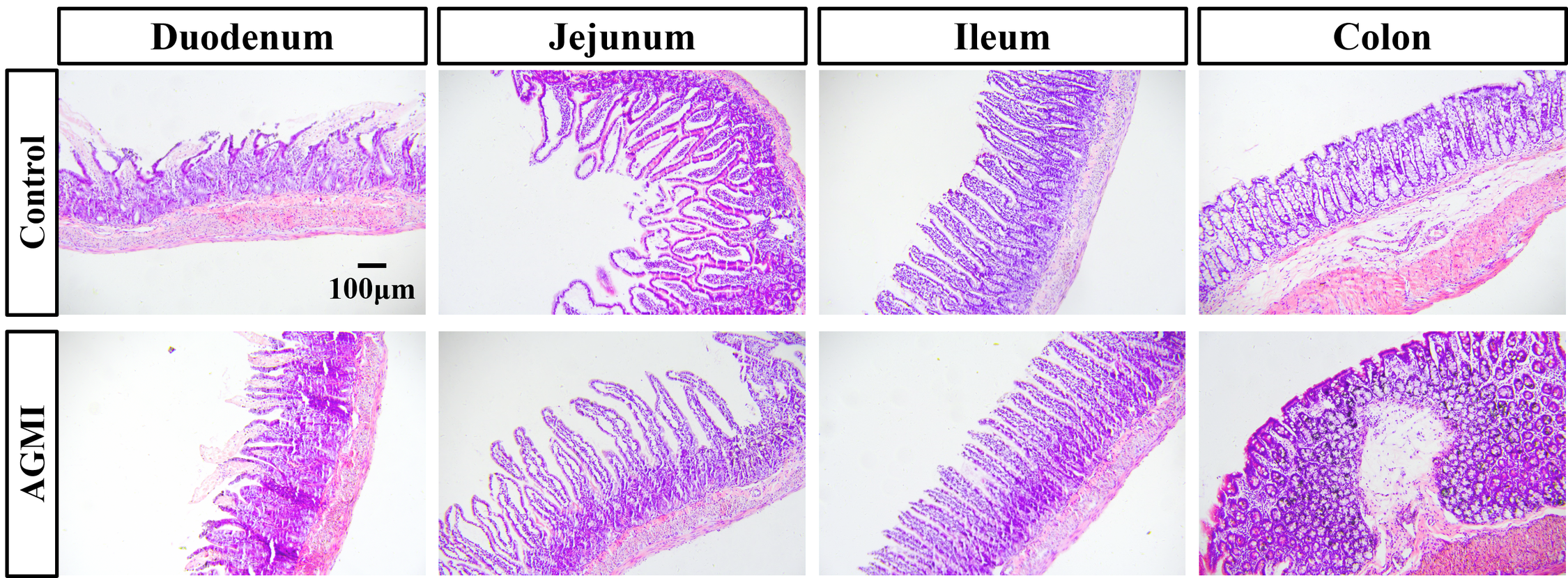

Supplement: S8 Fig — The duodenum, jejunum, ileum, and colon were inspected 13 hours post-modeling. Notably, the mucosal injury in the intestines appeared minimal following the modeling procedure (0.60 M). (TIF) [file pone.0324136.s008.tif]
